# The ΔF508-CFTR mutation inhibits wild-type CFTR processing and function when co-expressed in human airway epithelia and in mouse nasal mucosa

**DOI:** 10.1186/1472-6793-12-12

**Published:** 2012-09-24

**Authors:** Torry A Tucker, James A Fortenberry, Akos Zsembery, Lisa M Schwiebert, Erik M Schwiebert

**Affiliations:** 1 Departments of Cell Developmental and Integrative Biology, University of Alabama at Birmingham, 1918 University Blvd, Birmingham, AL 35294-0005, USA; 2 Gregory Fleming James Cystic Fibrosis (CF) Research Center, University of Alabama at Birmingham, 1918 University Blvd, Birmingham, 35294-0005 AL, USA; 3 Department of Experimental Human Physiology, Semmelweis University, Budapest, Hungary; 4 Department of Biochemistry, University of Texas Health Sciences Center at Tyler, Tyler, TX, USA; 5 DiscoveryBioMed, Inc, Birmingham, AL, USA

**Keywords:** Cystic fibrosis (CF), CFTR, Biogenesis, CF heterozygote, Oligomer, Chloride ion channels

## Abstract

**Background:**

Rescue or correction of CFTR function in native epithelia is the ultimate goal of CF therapeutics development. Wild-type (WT) CFTR introduction and replacement is also of particular interest. Such therapies may be complicated by possible CFTR self-assembly into an oligomer or multimer.

**Results:**

Surprisingly, functional CFTR assays in native airway epithelia showed that the most common CFTR mutant, ΔF508-CFTR (ΔF-CFTR), inhibits WT-CFTR when both forms are co-expressed. To examine more mechanistically, both forms of CFTR were transfected transiently in varying amounts into IB3-1 CF human airway epithelial cells and HEK-293 human embryonic kidney cells null for endogenous CFTR protein expression. Increasing amounts of ΔF-CFTR inhibited WT-CFTR protein processing and function in CF human airway epithelial cells but not in heterologous HEK-293 cells. Stably expressed ΔF-CFTR in clones of the non-CF human airway epithelial cell line, CALU-3, also showed reduction in cAMP-stimulated anion secretion and in WT-CFTR processing. An ultimate test of this dominant negative-like effect of ΔF-CFTR on WT-CFTR was the parallel study of two different CF mouse models: the ΔF-CFTR mouse and the bitransgenic CFTR mouse corrected in the gut but null in the lung and airways. WT/ΔF heterozygotes had an intermediate phenotype with regard to CFTR agonist responses in in vivo nasal potential difference (NPD) recordings and in Ussing chamber recordings of short-circuit current (ISC) in vitro on primary tracheal epithelial cells isolated from the same mice. In contrast, CFTR bitransgenic +/− heterozygotes had no difference in their responses versus +/+ wild-type mice.

**Conclusions:**

Taken altogether, these data suggest that ΔF-CFTR and WT-CFTR co-assemble into an oligomeric macromolecular complex in native epithelia and share protein processing machinery and regulation at the level of the endoplasmic reticulum (ER). As a consequence, ΔF-CFTR slows WT-CFTR protein processing and limits its expression and function in the apical membrane of native airway epithelia. Implications of these data for the relative health of CF heterozygous carriers, for CFTR protein processing in native airway epithelia, and for the relative efficacy of different CF therapeutic approaches is significant and is discussed.

## Background

CF is a monogenic disorder, a rare misfolded protein disorder, and the most common autosomal recessive genetic disease found in the Caucasian population [1-3]. CF is caused by mutations in CFTR that lead to reduced surface expression and/or function of this cyclic AMP-regulated chloride (Cl^-^) channel among other airway, gastrointestinal and other epithelial tissue defects [1-4]. The most commonly occurring CF mutation is the ΔF508-CFTR (ΔF-CFTR) mutation that occurs in approximately 70-90% of the CF population worldwide [1-4]. This mutation causes a folding defect in the CFTR protein that causes ER retention of the majority of the ΔF-CFTR protein [1-6].

CF disease phenotype correlates better with *CFTR* genotype in the gastrointestinal (GI) tract, where secretion of pancreatic enzymes and bile along with salt, bicarbonate, and water is essential for function [4]. However, in the CF lung and airways, there is little correlation between *CFTR* genotype and lung and airways disease phenotype [4]. One ΔF-CFTR homozygous patient can have severe disease and another ΔF-CFTR homozygous patient can present a more mild disease; this is the rationale for CF siblings and twins genotype/phenotype correlation studies currently in progress [7,8]. This lack of correlation may be explained by: (a) secondary or modifier genes that protect or fail to protect an individual from CF lung and airways disease progression [7]; (b) additional genes that cause predisposition to CF lung and airways disease progression [7,8]; and/or (c) CFTR’s known role as a regulator of other conductances and cellular processes [4]. Better understanding of ΔF-CFTR biology, physiology and lung and airways defects is critical, because the majority of the associated pathology and corresponding mortality of CF occurs in the pulmonary system.

One of the hypothesized and more viable methods to treat CF is by gene correction or protein replacement [9,10]. The goal is to introduce or replace the defective copy of CFTR with a functional wild-type (WT) copy that could generate a normal mRNA and a functional protein. Promising methods of introducing the WT-CFTR gene is via lipid- or virally-mediated transduction [9,10]. Barriers to these methods are currently being overcome [9,10]. One overwhelming problem is the lack of an animal model that displays the characteristic lung pathology seen in humans that a gene-bearing vector seeks to correct [9,10]; however, recent work on porcine and ferret animal models of CF is promising [11-13]. Work described herein introduces another concept that needs to be addressed in the context of these putative therapies: What if the mutant CFTR protein interacts with and affects the processing and function of the introduced WT-CFTR? A dominant negative-like effect of the endogenous ΔF-CFTR could also limit the effect of a WT-CFTR gene or protein correction or a CF corrector drug in a target cell.

Recent work has focused on examination of WT-CFTR and mutant CFTR biogenesis, trafficking, and functions within CFTR’s native environment, the polarized airway epithelial cell. In this light, we published important methods on transient transfection of CFTR into non-polarized and polarized epithelial cells [14]. We also showed that WT-CFTR processing in epithelial cells is more efficient than first suggested in heterologous cell systems over-expressing CFTR [15]. In the context of this work, we observed curious results that led us to test the hypothesis that mutant forms of CFTR can interact with and inhibit WT-CFTR function in airway epithelial cells. We present results herein with *in vivo* and *in vitro* approaches that support the hypothesis that ΔF-CFTR inhibits WT-CFTR in a dominant negative-like manner when co-expressed together in the same epithelium.

This hypothesis is germane to two different fields of CF research. The former relates to whether defects or predispositions to dysfunction are found in the CF heterozygous carrier. The latter involves whether or not CFTR exists within an oligomeric protein complex in epithelial cells as a monomer or a multimer. Throughout the clinical study of endpoints in CF, partial defects or dysfunction in the CF heterozygous carrier have been observed. However, because the CF carrier does not present with full progressive CF disease in the GI tract or in the lung and airways and because genotypes were not fully defined in these older studies, CF carriers have not be studied deeply or as a full study group compared to homozygotes or WT controls. Heterozygous cell models are also not available for similar reasons. However, partial loss in the volume of sweat or in rate of secretion in response to agonists has been documented in CF heterozygotes versus WT controls [16,17], whereas CF homozygotes fail to respond to agonists. Graded differences in sweat [Cl^-^ amounts were observed that yielded three statistically different groups in a continuum between CF homozygote patients, CF heterozygote carriers, and WT controls. Clinical endpoints have noted statistically valid predispositions to pancreatitis, rhinitis and sinusitis, allergic bronchopulmonary aspergillosis, and airway hyper reactivity in CF heterozygotes [18-21]. The latter predisposition to airway reactivity has been studied for several decades and have driven asthma geneticists to document prevalence of CF gene mutations in populations with severe asthma prevalence [18]. Additional studies were found in our literature review, but only the subset cited above studied all three genotypes. Nevertheless, the listed observations above provided a compelling rationale for studying wild-type CFTR and mutant CFTR interaction as a possible cause of heterozygote dysfunction.

A multitude of studies focusing on CFTR protein biochemistry have concluded that CFTR is a monomer [22-26]. However, this conclusion was supported by work largely performed in heterologous cell over-expression systems and was arrived at before the identification of CFTR binding partners at the N- and C-termini [27-34]. In particular, the identification of the PDZ-binding motif on the extreme C-terminal end of the carboxy-terminal tail and epithelial PDZ binding proteins such as EBP-50 (NHERF-1), E3-KARP (NHERF-2), CAP-70, and CAL among others have made many investigators re-think this conclusion [35-41]. This is particularly true in the context of the epithelial cell, where a single CFTR monomer could associate with a second CFTR monomer or with a larger number of CFTR monomers via PDZ-dependent contacts. Several investigators have shown that association with PDZ binding proteins affect CFTR Cl^-^ channel function, trafficking and localization [36-41]. Bear and colleagues have recently assessed the monomer versus multimer issue with CFTR expressed in different cell models and subjected the CFTR-enriched lysates to sucrose gradient analysis under non-denaturing and denaturing conditions [22]. Their conclusion was that CFTR existed as a monomer, as a dimer, and, possibly, in higher order multimers, but that a monomer was sufficient for Cl^-^ channel activity. Moreover, Naren and colleagues have shown in heterologous and epithelial cells that CFTR is a multimer that self-associates by a mechanism that does not appear to involve the PDZ motif in the C-terminus [23]. In addition, Cormet-Boyaka et al. characterized a trans-complementation mechanism where fragments of CFTR could rescue CFTR folding mutations [33]. They state that masking the mutated region of the CFTR polypeptide with a corresponding WT fragment could cause the mutant to escape the ER [32,33]. Zerhusen et al. showed that a CFTR concatemer acted in a similar manner to a single CFTR protein, arguing for possible cooperation of multiple CFTR proteins to form a functional channel [27]. In their discussion, these authors hint at the idea that mutant CFTR proteins could affect WT-CFTR [22,23,27,32,33]. The same has been studied recently for mdr P-glycoproteins, where monomeric and multimeric conclusions have been drawn [28]. Therefore, it is still an open question whether CFTR assembles as a multimer through: (a) self-association; (b) as an oligomeric complex; or (c) resides as a multimer within an oligomer. Nevertheless, there are compelling data from our laboratory and from others that multiple CFTR polypeptides can interact by either or both mechanisms in epithelial cells.

In the study herein, our data address both issues of heterozygote dysfunction and CFTR multimerization by assessing the dominant negative-like inhibition of WT-CFTR by ΔF-CFTR in human airway epithelial cells. Critically, the effect is specific to the ΔF-CFTR mutant. The results also speak to the need to overcome mutant CFTR effects on WT-CFTR introduced by emerging therapeutic methods. We show that ΔF-CFTR, when co-expressed with wild-type CFTR by multiple methods, inhibits WT-CFTR processing and, therefore, function in epithelial cells but not in heterologous cells.

## Methods and materials

### Cell culture

All cell culture substrates (plates, flasks, and filters supports) for epithelial cells were coated with 1:15 diluted Vitrogen 100 solution in Ca/Mg Free Dulbecco’s PBS (Life Technologies/Invitrogen). The diluted Vitrogen solution is added, allowed 2–3 min to coat the substrate, and is then removed for air drying in a sterile hood. CALU-3 (a human non-CF submucosal airway serous cell line endogenously expressing CFTR) [42] and HEK293T (a human embryonic kidney heterologous cell model over-expressing the large T antigen to amplify cDNA expression) [43] were grown in Minimal Essential Medium (MEM) with 10% heat-inactivated fetal bovine serum (Life Technologies/Invitrogen), 6 ml of penicillin-streptomycin 100× solution (penicillin 100 U/ml and streptomycin 100 μg/ mg final; Life Technologies/Invitrogen), 6 ml of 200 mM L-glutamine 100X solution (2 mM Final; Life Technologies/Invitrogen), and 2 ml of fungizone solution (amphotericin B, 1 ug/ml final; Life Technologies/Invitrogen). The IB3-1 cell line (derived from a CF human bronchus expressing the ΔF508 and W1282X mutant forms of CFTR) was grown in LHC-8 media without gentamycin (Biofluids) supplemented with 5% heat-inactivated fetal bovine serum, 6 ml of penicillin-streptomycin 100x solution (penicillin 100 U/ml and streptomycin 100 μg/mg final), 6 ml of 200 mM L-glutamine 100X solution (2 mM final), and 2 ml of fungizone solution (amphotericin B, 1 ug/ml final).

### Culture of polarized epithelial cell monolayers

CALU-3 non-CF human epithelial cells (parental, G418-resistant lacking mutant CFTR, and G418-resistant expressing mutant CFTR; see selection procedure below) were seeded onto coated 6.5 mm diameter polyester Transwell Filters (Corning-Costar, Corning, NY) at 1 × 10^6^ cells per insert. For these cell monolayers, a measured transepithelial electrical resistance (R_TE_) of > 2,000 Ω·cm^2^ was achieved routinely and sufficient to perform the subsequent Ussing Chamber transepithelial Cl^-^ secretion assays.

### Transient transfection of non-polarized epithelial cells

These methods have been published previously [14]. However, co-transfection of wild-type and mutant CFTR cDNAs was a novel feature of this study to simulate a “heterozygous” cell. The methods of LipofectAMINE PLUS-mediated transient transfection were similar; however, the DNA combinations were varied in the following manner for a typical experiment presented below for cells grown in a 10 cm diameter coated culture plate:

• EV or Empty Vector = 6.75 μg of pcDNA 3.1 plasmid DNA devoid of CFTR cDNA

• WT-CFTR-bearing Vector = 0.75 μg (Balance “backfilled” with 6 μg of empty vector or EV)

• ΔF-CFTR-bearing Vector = 0.75 μg (Balance “backfilled” with 6 μg of EV).

Note below that the amount of ΔF-CFTR was increased in a titration to determine how much ΔF-CFTR vector needed to be transfected to make ΔF-CFTR protein that was equivalent to WT-CFTR because of the dramatically reduced protein half-life of this ER retention mutant [44,45]. Thus, in other transiently transfected cultures, a mixture of WT-CFTR and ΔF-CFTR-bearing vector was co-expressed in the same cells in the following mixtures:

• 1×WT with 1×ΔF = 0.75 μg WT, 0.75 μg ΔF, 5.25 μg EV

• 1×WT with 2×ΔF = 0.75 μg WT, 1.5 μg ΔF, 4.5 μg EV

• 1×WT with 4×ΔF = 0.75 μg WT, 3.0 μg ΔF, 3.0 μg EV

• 1×WT with 8×ΔF = 0.75 μg WT, 6.0 μg ΔF; 0 μg EV

These ratios were used for the IB3-1 CF and HEK-293 T cells transfected transiently. For G551D-CFTR experiments, the same amounts of G551D-CFTR bearing plasmid were used as a substitute for ΔF-CFTR. These DNA combinations were incubated with PLUS reagent in OptiMEM-1 serum-free medium for 15 min at room temperature. After the first incubation, LipofectAMINE reagent from a separate tube was mixed with the PLUS reagent-primed plasmid DNA combinations. The complete transfection cocktail was incubated for another 15 min at room temperature. During the incubation periods, the cells were washed 3X with Opti-MEM-1 medium to remove all serum and to sensitize the cells to the serum-free medium. After the final wash, the transfection cocktail was brought up to a final volume of 6 mls from a mixing volume of 1 ml. The cells were then incubated for 6 h at 37°C in the humified CO_2_ incubator. After the 6-h incubation, the cells were washed 2× with Opti-MEM and 1× with FBS containing media to remove excess lipid-DNA complexes. The cells were re-fed 24 h after transfection and studied for CFTR biochemistry and function 48 h post-transfection.

### Transient transfection of HEK293T heterologous cells

Similar methods were followed to those described above with the notable exception that Effectene reagent (Qiagen) was used for HEK293T cells [14]. This reagent was found to be toxic to all epithelial cell models but ideal for HEK-293 cells [14]. Surprisingly, there was minimal toxicity to HEK-293 cells while a transfection efficiency of 90-95% was routine. Enhancer reagent was added to OptiMEM-1 medium along with the same DNA combinations above. The mixture was incubated for 10 min at room temperature. After the initial incubation, 24 μl of Effectene reagent was added to each tube, followed by 10 min incubation at room temperature. During the incubations, the cells are washed 3× with Opti-MEM. After the final wash, all media is removed from the cells, and transfection cocktails are brought up to a 6 ml volume and added to the culture dishes. The cells were incubated in transfection cocktail for 4 h at 37°C in the humified CO_2_ incubator. After the 4-h incubation, the cells were washed 2× with Opti-MEM and 1× with FBS containing media. The cells were re-fed 24 h after transfection and studied for CFTR biochemistry and function 48 h post-transfection.

### Stable transfection and selection of “heterozygous” cells

Similar LipofectAMINE PLUS-based methods were used as above. Vector bearing ΔF508-CFTR cDNA was transiently transfected in combination with the pcDNA 3.1 vector with a G418-resistance gene cassette to confer antibiotic resistance into the non-CF airway epithelial cell lines, CALU-3. CALU-3 cells grow as ‘islands’ of cells that eventually grow and fuse together as a confluent monolayer. The cells were transfected as small islands dispersed throughout the culture dish. The cells were re-fed 24 h after transfection and cultures were allowed to grow until the islands grew much larger but were still not yet fused together as a confluent culture. After 7–10 days of culture as described above, MEM complete media was added that was also supplemented with 700 μg/ml of genetic in (G418) to select stably transfected CALU-3 cell islands. The cells were washed with PBS and fed G418-containing MEM complete medium every other day that was made fresh and filtered to keep G418 activity high in the cultures. The majority of the cell islands died; however, some cells within islands lived and began to form isolated colonies. These ‘island colonies’ were then selected using cloning rings (the reason for using 10 cm diameter dishes was that sterile cloning rings could be inserted by simply lifting the lid of the dish). The cloning rings were dipped in sterile, autoclaved vasoline gel to allow them to adhere to the bottom of the plate. Once these colonies grew to confluence within the cloning ring, the clonal ‘island colony’ was transferred to a 24 well plate for further expansion. They were then expanded further into flasks as well as frozen in micro-aliquots to have the earliest possible passage following selection cryopreserved.

### Immunoprecipitation and phospholabeling of CFTR

Published methods were followed [15]. Cells were washed 1X with CaMg containing PBS. The cells were kept at 4°C during washes. The cells were subsequently lysed in “Radioimmunopreciptiation Assay” (RIPA) Buffer containing NP40 (1%), sodium deoxycholate (.5%), SDS (0.1% at pH 8.0), and sodium chloride (150 mM) supplemented with Protease Inhibitor Cocktail (Roche). Samples were homogenized and incubated for 30 min at 4°C. The lysates were then centrifuged at 14,000 g at 4°C for 20 min. The supernatants were then collected and protein concentrations calculated using the BCA Protein Assay Kit and a microplate reader. Immunoprecipitations were performed using Protein A Agarose and the anti-CFTR Ab targeted to the NBD-1/R region of CFTR (Bedwell/Collawn) with at least 800 ng of lysate supernatant. The supernatants were added to the Ab/Protein Agarose Solution and allowed to incubate at 4°C for 2 h or overnight on an end-over-end shaker (rotator). Samples were then centrifuged at 14,000 g for 2 min and the supernatant removed from the pelleted agarose beads. RIPA buffer (750 μl) was then added to the beads, centrifuged for 2 min at 14,000 g, and the supernatant removed. This was repeated 2 times. On the final wash, the pelleted beads were washed with 750 μl PKA Buffer. Two μl of PKA catalytic subunit and 10 μl [γ-^32^P] ATP were added to phosphorylate the bound CFTR to make it detectable with phosphor-imaging technology. The samples were then allowed to incubate for 45 min at 30°C. After the incubation, the cells were washed 3X with RIPA buffer at room temperature. Excess RIPA buffer was then removed from the beads. Thirty-five μl of 2X sample buffer with β-mercaptoethanol was added to the samples and incubated at 37°C for 15 min. Samples were then run on a 6% Tris Agarose gel at 150 V for 90 min, dried in a gel dryer, and analyzed on the PhosphorImager.

### Voltohmeter open-circuit and ussing chamber short-circuit current measurements of monolayer electrical properties

R_TE_ was measured using the Millipore MilliCell ERS Voltohmeter that uses Ag-AgCl pelleted chopstick electrodes. The R_TE_ was monitored on a daily basis and was used as an indicator of the level of maturity of the monolayer. When monolayers had matured and reached a R_TE_ plateau (> 1,000-2,000 Ohms), Ussing chamber recordings of the short-circuit currents were performed. Ussing chamber experiments were performed as described previously [46]; however, they were designed to activate and monitor CFTR Cl^-^ currents in accordance with recent published studies. Recordings were performed in OptiMEM-1 reduced serum medium or in Ringers enriched with bicarbonate on CALU-3 non-CF airway epithelial cells grown as monolayers (a submucosal gland serous cell model with abundant endogenous CFTR expression) that were or were not stably transduced with ΔF508-CFTR. Amiloride (10 μM) was added to the apical solution to inhibit any residual ENaC-mediated Na^+^ currents which were negligible in these cell models grown under these conditions. Then, forskolin (2 μM) was added to both sides of the cell monolayers to increase cyclic AMP and stimulate CFTR Cl^-^ conductance. To maximally activate CFTR Cl^-^ conductance in these monolayers, genistein (50 μM) was added to both sides of the monolayer. In some experiments, glibenclamide (glyburide, 50 μM) was added to inhibit the CFTR-mediated Cl^-^ conductance (data not shown). Magnitude of the forskolin- and genistein-activated Cl^-^ conductance was compared statistically between parental or G418-resistant clones CALU-3 cells that lacked ΔF508-CFTR versus those that possessed ΔF508-CFTR.

### SPQ halide fluorescence assay of halide transport

The SPQ assay was used to detect the amount of active CFTR Cl^-^ channels that are functional at the plasma membrane of the transiently transfected cells. It has been used previously by our laboratory in published papers [47]. The SPQ fluorescent dye is sensitive to halides, some of which quench the dye’s fluorescence (iodide, chloride) and some of which do not (nitrate). The cells are seeded onto a glass coverslip. After a 24 h period, the cells were then transiently transfected with the CFTR cDNAs. Twenty-four hours after initiation of transfection, the cells were placed in media containing the SPQ dye (10 mg/ml) for overnight incubation. After another 24 h, the cover slips were taken and placed on the fluorescence scope (the cover slips actually form the bottom of the flow chamber). Twenty-five to 30 individual cells were selected based upon the intensity of their fluorescence, which denotes the efficiency of SPQ dye uptake. Their fluorescence is then measured and recorded. The SPQ fluorescence protocol is as follows. The cells were placed in the perfusion chamber and exposed to 3 different buffers: (1) NaI buffer (iodide enters the cells and quenches SPQ fluorescence; (2) NaNO_3_ buffer (nitrate reverses this gradient and allows the iodide to passively diffuse out of the cells and unquenches the fluorescence); (3) NaNO_3_ buffer with a cAMP agonist cocktail (100 μM isobutylmethylxanthine (IBMX), 10 μM forskolin, and 200 μM dibutyryl-cAMP, 8-bromo-cAMP or CPT-cAMP) to stimulate CFTR Cl^-^ conductance and stimulate additional iodide efflux from the cell; and (4) NaI buffer without agonists, to wash out and reverse agonist effects as well as re-quench SPQ fluorescence. The reversibility and re-quenching is also a good indicator of the viability of and level of dye within the cells throughout the entire experiment. The relative background for each cover slip was subtracted from the recorded arbitrary light unit measurements. The resultant data points are analyzed and plotted dependent upon the first recorded point which establishes the baseline.

### Nasal potential difference (NPD) assays on two different cf mouse models

NPD assays were performed as described previously but were designed to specifically study and activate CFTR maximally. We performed experiments on all three *CFTR* genotypes in each mouse model. One mouse model was the ΔF508-CFTR mouse developed by Thomas and colleagues [48]. The second mouse model was the UNC knockout mouse that was corrected in the gastrointestinal tract by complementation with a fatty acid binding protein (FABP) promoter-driven *CFTR* construct. The lung and airways remain null for CFTR (a generous gift from Dr. Jeffrey Whitsett, M.D., Ph.D. to the UAB CF Center, [49]. Amiloride (50 μM) was added in a standard Lactated Ringers in step 1 of the assay to inhibit all Na^+^ absorptive pathways. In the continued presence of amiloride, a low Cl^-^ (6 mM) solution was perfused to gauge Cl^-^ permeability of the nasal mucosa epithelium. Wild-type and heterozygous animals possessed a low Cl^-^ response, while homozygous animals did not. In the presence of amiloride and in a low Cl^-^ solution, adenosine and salbutamol (100 μM each) were added together to increase cyclic AMP maximally via their respective G protein-coupled receptors that engage adenyl cyclase. Isoproterenol (Isoprel™) or albuterol (salbutamol) alone were not enough to maximally stimulate CFTR. It was adenosine together with a β-adrenergic agonist that gave us consistent cyclic AMP induction of CFTR above and beyond the low Cl^-^ response. This modification was done based on the work of Clancy and colleagues on adenosine regulation of CFTR in mice and humans [50,51]. The change in PD, in the negative direction (hyperpolarization) during the low Cl^-^ phase and adenosine and salbutamol (cyclic AMP) phase of the recordings, was quantified from the strip-chart records. Ussing chamber assays were performed as described above on primary mouse tracheal epithelial cell monolayers derived from these mice.

### MTE monolayer culture and ussing chamber analysis

After analysis of *in vivo* CFTR function by nasal PD in the three *CFTR* genotypes in each mouse model, tracheae were excised by surgery in anesthetized mice and were kept separated as to genotype. Tracheae were first washed in a CaMg-free PBS with 5X penicillin/streptomycin (500 U/ml penicillin, 500 μg/ml streptomycin). Tracheae were then washed in a series of dishes containing CaMg-free DMEM/F12 medium with 5X penicillin/streptomycin. Excess tissue was removed from the tracheae, and they were filleted open down the midline from the laryngeal cartilage to its base. The dissection was performed in the above DMEM/F12 Dissection Medium. The filleted tracheae were then placed in CaMg-free DMEM/F12 medium with 2X penicillin/streptomycin and 1 μg/ml protease type XIV and 0.1 μg/ml DNase I on ice. The tracheae were digested at 4°C overnight in this Digestion Medium without agitation. After the 18 h overnight digestion in the cold, tracheae were inverted in the tubes 15X to maximally dissociate cells. FBS (20%) was then added to inactivate the enzymes, and the dissociated cells were placed into a separate tube on ice. The digested tracheae were then washed (with 15X tube inversion again) in mouse tracheal epithelial (MTE) cell Monolayer Maturation - CaMg-containing DMEM/F12 medium supplemented per 500 mls of medium with 20% FBS (Life Technologies, Certified and Heat-Inactivated), 2X Pen/Strep, 5 mls of L-glutamine (from 100X stock), 2 mls of hydrocortisone (from 10 ml stock solubilized in ethanol, Becton-Dickinson), 2 mls of endothelial cell growth supplement (ECGS from 10 ml stock, Becton-Dickinson), 2 mls of bovine pituitary extract (BPE from 10 ml stock, Becton-Dickinson), and 4 mls of insulin-transferrin-selenium (ITS from 20 ml stock, Becton-Dickinson). Washes were collected and placed on ice. Then, the tracheae were exposed to fresh Digestion Medium for 1 h at 37°C. After the second digestion, the tubes were inverted 15X, FBS (20%) was added to inactivate the enzymes, dissociated cells were collected in a separate tube, and washes with standard culture medium were performed as above. Four separate sets of tubes (two digestions, two washes) were centrifuged for 3–5 min to pellet any and all cells. Pellets were then obtained from all 4 phases of the MTE cell isolation. Then, cells were combined (still separated as to *CFTR* genotype), pelleted and resuspended in a minimal volume (50–100 μl per filter support). Four filter supports can be seeded per 1 trachea. Filter supports were coated with CellTak and with a 1:10 diluted Vitrogen 100 solution (in CaMg-free PBS) that also contained 1 mg human fibronectin (Becton-Dickinson) added into a 50 ml total volume one day prior to seeding. The cell seeding day was deemed Day 0. Cells were allowed to attach over an initial 3-day period with medium bathing both sides of the filter support. Then, on Day 3, medium was removed and only the basolateral (bottom) side of the filter support was fed to initiate air-fluid interface (AFI) culture. MTE monolayers were maintained in this way until leak of medium was no longer observed from the bottom to the top of the filter support. Visual inspection of the cells on the filter support when no leak was observed showed “doming” and “ridging” of a confluent monolayer. After this point in monolayer culture, R_TE_ and V_TE_ were monitored with a Voltohmeter (Millipore or World Precision Instruments). R_TE_ above 1,000 Ω·cm^2^ and a significant negative V_TE_ were then measured on or after Days 8–10. We performed Ussing chamber analysis when the electrical parameters had plateaued in open-circuit measurements and did not increase further.

#### Statistics

Explanation of quantification and statistical analysis of the data generated in all assays was explained in the context of the specific methods presented above.

## Results

### Early Evidence of ΔF508-CFTR inhibition of wild-type CFTR function

As a collaborative effort among multiple authors and laboratories involved in this study, a study was published in which optimization of transient transfection of polarized epithelial cell monolayers was performed [14]. The founding context of this work was that CFTR biogenesis, trafficking and function would be best studied in its native environment, the polarized human airway epithelial cell. During these studies, we observed that ΔF-CFTR expression in epithelia inhibited WT-CFTR driven cyclic AMP-activated Cl^-^ channel activity, monolayer maturation, and regulation of chemokine release. These observations provided the rationale for designing and undertaking the studies described below.

### Is the expression of wild-type cftr altered by co-expression of ΔF508-CFTR?

**Figure 1:**
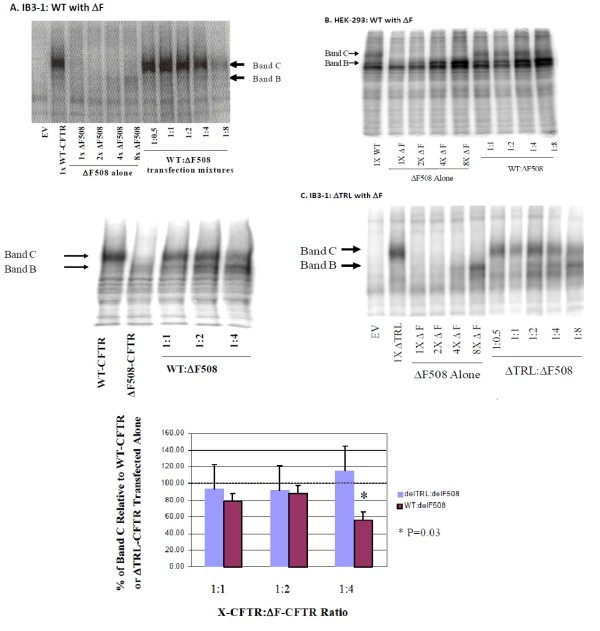
**Co**-**expression of increasing amounts of ΔF**-**CFTR alters the processing of WT**-**CFTR but not** Δ**TRL**-**CFTR in IB3**-**1 CF epithelial cells.** ΔF-CFTR does not influence WT-CFTR in heterologous human HEK293T cells. **A**. IB3-1 CF bronchial epithelial cells transfected transfected with a fixed amount of WT-CFTR and increasing amounts of ΔF-CFTR. Two different examples are shown, a full titration and a partial experiment imaged by different methods. **B**. HEK293T cells were transfected with the same mixtures and CFTR detected biochemically. **C**. The relative effect of ΔF-CFTR on the processing of a fixed amount of ΔTRL-CFTR in the presence of increasing amounts of ΔF-CFTR provided in a typical blot and effect of ΔF-CFTR on both fully processed CFTR constructs (WT and ΔTRL) was quantified and graphed.

To determine whether the processing of WT-CFTR is affected by the presence of ΔF-CFTR, we co-expressed the WT and mutant forms of CFTR in IB3-1 CF human airway epithelial cells that are null for detectable endogenous CFTR protein (Figure 1A). Examination of immunoprecipitated and PKA-decorated proteins on a 6% SDS-PAGE gel showed that processing of a fixed amount of WT-CFTR was altered by increasing amounts of ΔF-CFTR. In native epithelia, CFTR is immunoprecipitated as two major forms. “C band” is a broad band between 160–180 kDa that is the maturely glycosylated form of CFTR that successfully traffics through the secretory pathway to the apical plasma membrane. C band is the only form found when exogenous WT-CFTR was expressed alone in IB3-1 cells. ”B band” is a tighter immaturely glycosylated band between 140–150 kDa that is an ER form of CFTR. It is a single band in native epithelia and a doublet of bands in HEK-293 cells (see below). B band is the only band observed when exogenous ΔF-CFTR was expressed alone IB3-1 cells. In Figure 1A, as the amount of ΔF-CFTR cDNA was increased in the presence of a fixed amount of WT-CFTR cDNA, there was decreased processing of the C band of WT-CFTR protein. Inhibition of C band formation was most notable when 4-8-fold more ΔF-CFTR plasmid was co-transfected versus WT-CFTR (refer to both examples in Figure 1A for different examples of the same dominant negative-like effect. We speculate that this is due to the increased rate of degradation in the ER of ΔF-CFTR versus WT-CFTR [44,45]. However, when the same co-transfection experiment was performed in the heterologous human embryonic kidney cell line, HEK-293 T, over-expression of ΔF-CFTR was without effect on WT-CFTR processing (Figure 1B). These results suggest that CFTR processing may be different in epithelial cells versus heterologous cells and that epithelial-specific accessory proteins may be essential for driving this ΔF-CFTR inhibitory interaction with WT-CFTR.

### Is this dominant negative-like effect of ΔF508-CFTR on WT-CFTR dependent upon Its PDZ motif?

To assess specificity of this ΔF-CFTR/WT-CFTR interaction, we co-expressed ΔF-CFTR with a fixed amount of ΔTRL-CFTR, a CFTR variant lacking the C-terminal PDZ binding motif but that processes efficiently through the Golgi to the plasma membrane [52]. The CFTR PDZ motif is critical for CFTR interactions with PDZ binding proteins such as EBP50 (NHERF), E3KARP, CAP-70, CAL, etc., connects CFTR to larger macromolecular complexes in organelle and plasma membranes, and influences CFTR function [35-41,53]. In contrast to dominant negative-like effects on WT-CFTR by ΔF-CFTR, increasing amounts of ΔF-CFTR failed to affect the maturation of ΔTRL-CFTR in IB3-1 CF human airway epithelial cells (Figure 1C). Summary data is also presented from multiple experiments (Figure 1C). ΔF-CFTR was also without effect on ΔTRL-CFTR in HEK-293 T cells (data not shown). These results suggest that the PDZ motif of CFTR is critical to the inhibitory influence of ΔF-CFTR on WT-CFTR at the level of the ER.

### Is this a specific and exclusive interaction between ΔF508-CFTR and WT-CFTR?

**Figure 2:**
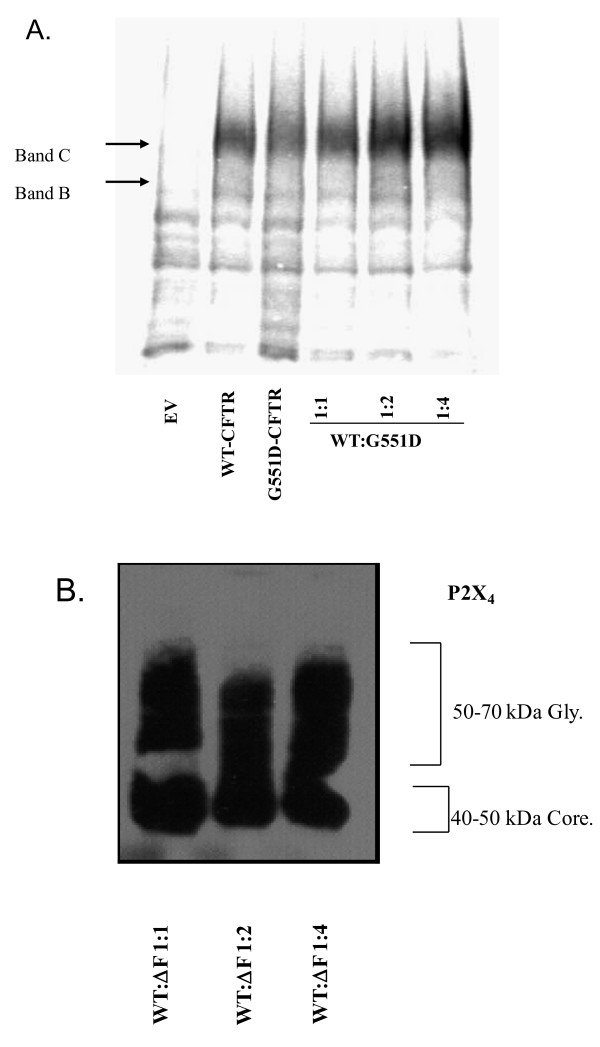
**Confirming the specificity of the dominant negative**-**like effect of** Δ**F**-**CFTR on WT**-**CFTR in native IB3**-**1 CF bronchial epithelial cells. A**. Similar co-expression biochemical experiments with G551D-CFTR and WT-CFTR. **B**. Increasing amounts of ΔF-CFTR do not affect the maturation and processing of a different glycosylated membrane protein, the purinergic receptor channel, P2X4.

We wished to take addition steps to provide specificity and exclusivity for this macromolecular complex and PDZ motif-driven ΔF-CFTR/WT-CFTR inhibitory interaction, we expressed increasing amounts of G551D-CFTR with a fixed amount of WT-CFTR. We observed only accumulating amounts of C band that did not appear to saturate during the co-expression of this mutant and WT-CFTR (Figure 2A). It is important to underscore the fact that G551D-CFTR is not an ER retention mutant but rather is processed normally to the plasma membrane. Rather, G551D-CFTR is a dysfunctional Cl^-^ channel because ATP binding and gating to its NBDs is impaired. This is why the CFTR potentiator drug, VX-770 (ivacaftor, Kalydeco) [54] is markedly effective in G551D-CFTR patients, while the CF corrector drug, VX-809, corrects/rescues ΔF-CFTR from ER quality control and is without effect on G551D-CFTR patients [55,56]. We also wished to determine whether this dominant negative-like effect of ΔF-CFTR was not non-specific to other glycosylated membrane proteins. The epithelial P2X_4_ purinergic receptor calcium entry channel also has immature (~40 kDa) and maturely glycosylated (60–65 kDa) forms and robust expression in both CF and non-CF human airway epithelial cells [57]. P2X_4_ expression is robust in Western blot analysis and no difference in expression of either form of the receptor channel was observed with increasing amounts of ΔF508-CFTR plasmid expression (Figure 2B). These data, along with other internal controls above in Figure 1, suggest that ER stress is not a cause of ΔF-CFTR inhibition of WT-CFTR processing.

### Is the function of WT-CFTR altered by co-expression of ΔF-CFTR?

**Figure 3:**
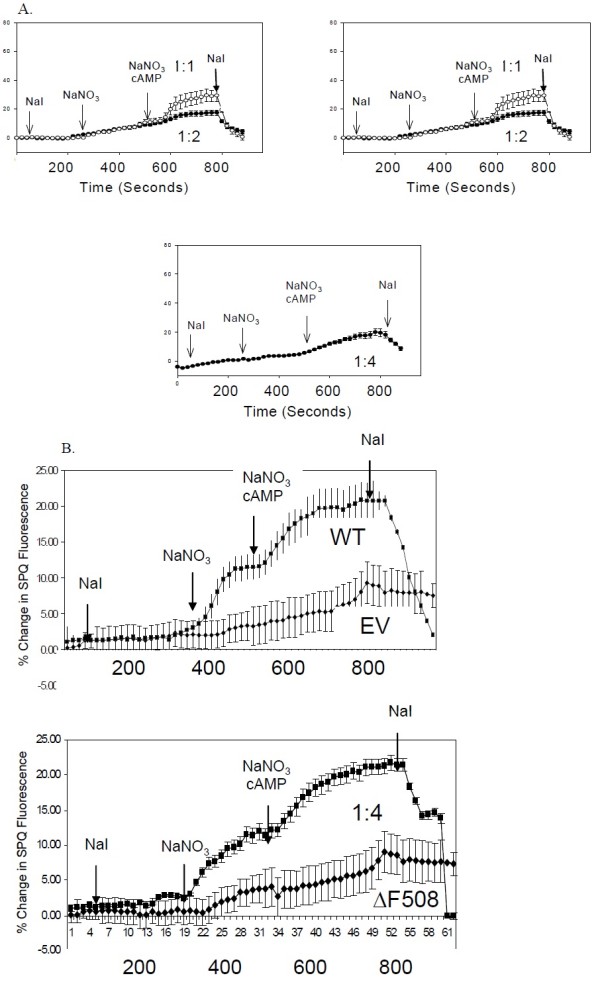
**Co**-**expression of increasing amounts of** Δ**F**-**CFTR alters the function of WT**-**CFTR in IB3**-**1 CF epithelial cells but not in HEK293T cells. A**. IB3-1 cells are seeded on Vitrogen coated coverslips and transiently transfect in the manner previously described in Figure 1. Twenty-four hours after transfection the cells are loaded with the halide sensitive dye, SPQ, overnight. The cells relative fluorescence is then measured while incubated in three different buffers: NaI, NaNO_3_, and NaNO_3_ with cAMP agonists, and then back into NaI. **B**. HEK293T cells were seeded on Vitrogen free coverslips and transfected, loaded, and measured as previously described in **A**.

To complement the biochemical experiments above, we also assayed for CFTR Cl^-^ channel function with the SPQ halide efflux assay [47]. IB3-1 cells were co-transfected with increasing amounts of ΔF-CFTR versus a fixed amount of WT-CFTR as above. These cells were later incubated with the halide-sensitive dye, SPQ, overnight in medium prior to the experiment 2 days after transient transfection. Cover slips of transiently transfected cells were mounted into a perfusion chamber and bathed in sodium iodide (NaI) buffer to maintain quenching of SPQ fluorescence. First, the cells were challenged with NaNO_3_ buffer, which dequenchs SPQ and assays for basal halide efflux which is augmented by WT-CFTR expression. Second, a cocktail of cyclic AMP agonists (CPT-cAMP, 200 μM; forskolin, 2 μM; IBMX, 100 μM) was perfused into the chamber in NaNO_3_ buffer to stimulate additional CFTR Cl^-^ channel activity (measured by increased halide efflux). Mock and ΔF-CFTR-expressing cells failed to respond to NaNO_3_ buffer alone or to the cocktail of cyclic AMP agonists (Figure 3A). In contrast, WT-CFTR-transfected cells responded markedly to both NaNO_3_ buffer alone or to the cyclic AMP cocktail (Figure 2A). However, in the co-expression experiments, increasing amounts of ΔF-CFTR inhibited wild-type CFTR activity in an apparent dose-dependent manner (Figure 3A). The functional assay showed complete inhibition with 4:1 ΔF-CFTR:WT-CFTR expression, while biochemical assays showed complete inhibition with 8:1 ΔF-CFTR:WT-CFTR expression. In contrast, similar experiments in the HEK-293 T system showed no inhibition of WT-CFTR with increasing amounts of ΔF-CFTR (Figure 3B). Taken together, these data suggests that ΔF-CFTR interacts with WT-CFTR during its processing and inhibits its functional expression in the plasma membrane in a dominant negative-like manner in human airway epithelial cells.

Are the processing and function of WT-CFTR altered by stable expression of ΔF-CFTR in a polarized non-CF human airway epithelial cell line that expresses endogenous CFTR?

**Figure 4:**
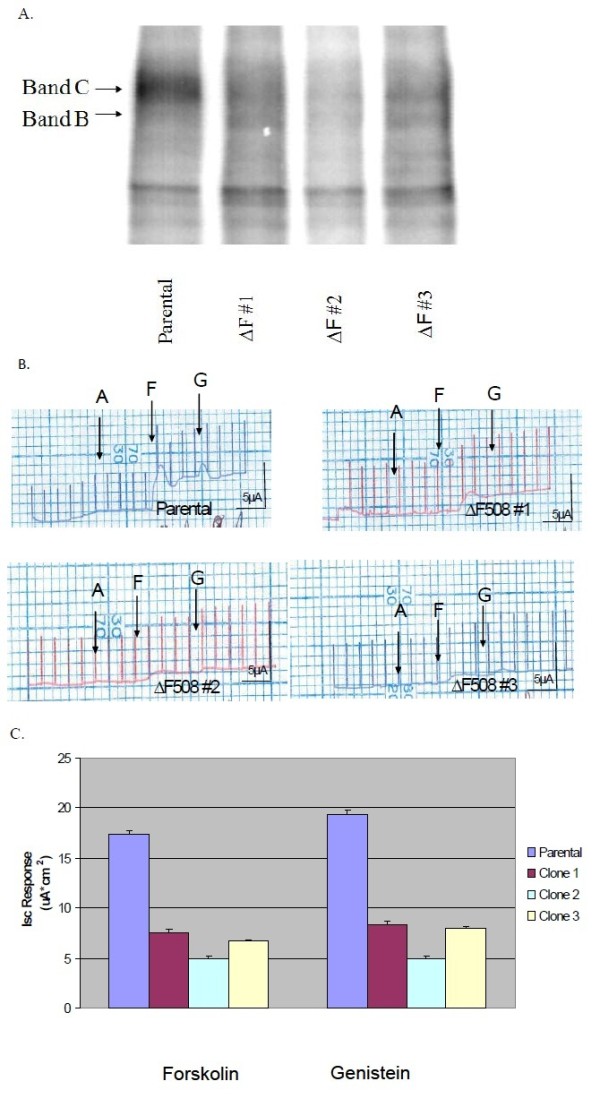
**Stable Transfection of** Δ**F508**-**CFTR into CALU**-**3 non**-**CF epithelial cells alters the processing and function of endogenous WT**-**CFTR. A**. CFTR was IP’d from parental and stably transfected CALU-3 cells. The samples were then phosphorylated and resolved as described previously. **B**. Parental and stably transfected CALU-3 cells were seeded on 6.5 mm Vitrogen coated permeable filter supports. The CALU-3 monolayers were then allowed to reach a transepithelial resistance (R_TE_) of 2,000 Ω·cm^2^ or above prior to experimentation. I_SC_ is then measured in response to 10 μM amiloride, 20 μM forskolin and 50 μM genistein added apically via an Ussing chamber. Typical traces are shown. **C**. Summary analysis of parental and stably transfected CALU-3 cells I_SC_ response to agonists.

One potential problem of the co-transfection and co-expression studies above was the necessity to transiently transfect with large quantities of plasmid DNA and to over-express ΔF-CFTR in order to inhibit WT-CFTR processing and function. Again, we speculate that this is likely necessary to overcome the increased rate of degradation of ΔF-CFTR protein versus WT-CFTR protein. However, to account for this issue and to approach this inhibitory interaction differently, we employed the well-characterized WT-CFTR-expressing airway epithelial cell line, CALU-3, and stably transfected ΔF-CFTR into it, generating several clones that were CF heterozygous cell lines. We also chose the CALU-3 cell line because of its high level of endogenous WT-CFTR expression and its ability for form polarized cell monolayers when grown on filter supports. Upon expansion and cryopreservation of the clones, CFTR biochemistry was performed. Parental CALU-3 cells expressed the C band form of CFTR almost exclusively (Figure 4A); all clones stably expressing ΔF-CFTR as an engineered heterozygous CF cell expressed both B band and C band forms and with reduced C band amounts in all clones. To assay for CFTR Cl^-^ channel function, we grew parental CALU-3 cells and ΔF-CFTR-expressing CALU-3 clones on permeable filter supports for Ussing chamber analysis of short-circuit current (I_SC_). Monolayers with a transepithelial resistance (R_TE_) at or above 1,000 Ω·cm^2^ were used in these experiments. In all experiments (Figure 4B and C), amiloride (10 μM) was added to block any Na^+^ absorption which is minimal in this epithelial cell model. Typical traces are shown for a parental CALU-3 cell monolayer and for multiple stable clones expressing ΔF-CFTR as an engineered heterozygous cell model co-expressing WT-CFTR and ΔF-CFTR endogenously. After amiloride pretreatment, forskolin (10 μM) was added to stimulate CFTR-dependent Cl^-^ secretion via cyclic AMP. Then, genistein (50 μM) was added to open any and all remaining CFTR Cl^-^ channels in the apical membrane. While parental CALU-3 cell monolayers responded with an averaged 5 μA to forskolin and an additional 1 μA to genistein in the presence of forskolin (Figure 4B), stable clones co-expressing both WT-CFTR and ΔF-CFTR endogenously responded only half as well or less so than parental cell monolayers. Figure 4C provides the summary data for this Ussing chamber analysis. Taken together, these data show that equivalent expression of WT-CFTR and ΔF5-CFTR endogenous to an airway epithelial cell leads to inhibition of WT-CFTR processing and, thus, function. Stable expression of both forms of CFTR also obviated the need to express more ΔF-CFTR in a transient transfection versus WT-CFTR to observe the same dominant negative-like effect.

### Is the function of WT-CFTR altered by ΔF-CFTR in WT-CFTR/ΔF-CFTR heterozygous ‘carrier’ mice in vivo and in vitro?

*In vitro* results above suggested a dominant negative-like inhibition of WT-CFTR by ΔF-CFTR that was specific to this most common ER retention folding mutant and observed in native human airway epithelial cells. Studies of human patients populations, where the WT (normal), heterozygous carrier, and homozygous CF patients were analyzed as separate groups, has shown three different phenotypes for a given endpoint in past studies. We wished to confirm our *in vitro* studies with *in vivo* nasal potential difference (NPD) measurements in the ΔF508-CFTR mouse [48]. A previous argument explaining partial CF heterozygous defects was simply gene dilution (e.g., 1 copy or allele of WT-CFTR versus 2 copies). As such and in parallel, we performed NPD assays on a bitransgenic CF mouse model generously provided to our UAB CF Center Mouse Transgenic CORE by Dr. Jeffrey Whitsett. This model is a CFTR knockout mouse that is corrected in the gastrointestinal tract with a fatty acid binding protein (FABP) promoter-driven *CFTR* construct [49]. In this bitransgenic mouse, however, the lung and airways remain null for *CFTR* in the CF (−/−) homozygous condition, the heterozygous mice have 1 allele of CFTR (+/−), and the WT mice have two alleles of CFTR (+/+). This is different from the ΔF-CFTR mouse, where the WT mice will be WT/WT, the heterozygotes will be WT/ΔF, and the homozygotes will be ΔF/ΔF.

**Figure 5:**
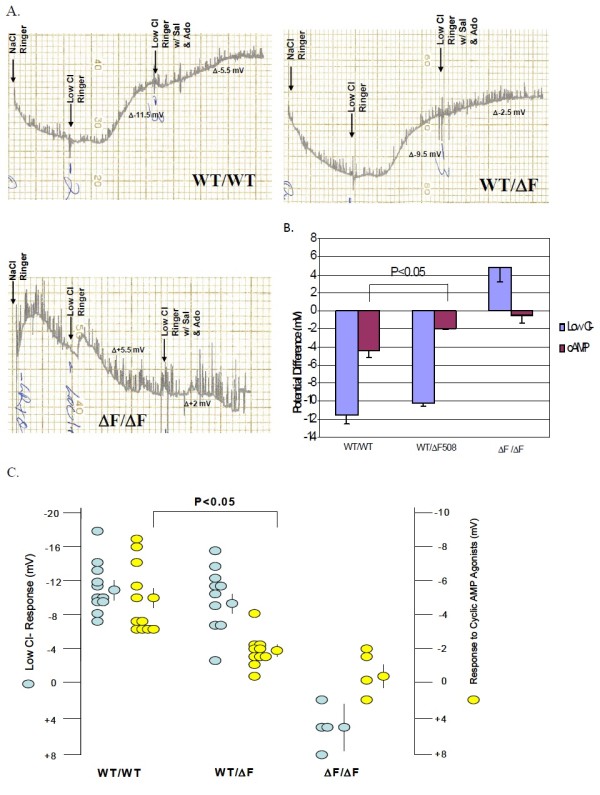
**Nasal Potential Difference is altered by ** Δ**F508**-**CFTR in WT**- **CFTR**/Δ**F508**-**CFTR Heterozygous mice. A**. Nasal Potential Difference Assays (NPD) were performed on the three genotypes of the ΔF508 mice: WT/WT, WT/ΔF, and ΔF/ΔF. NPD recordings were measured for each genotype in the presence of normal Ringer’s solution, low Cl- ringer’s solution, and then low Cl- Ringer’s solution containing the cAMP agonist salbutamol (100 μM) and adenosine (100 μM). **B**. Summary data of the results illustrated in **A**. **C**. Scatterplot representation of the NPD measurements performed in the 3 genotypes of the ΔF508 mice.

Figure 5A shows typical NPD recordings from all 3 genotypes of the ΔF-CFTR mouse. Summary data that includes and illustrates results from all mice studied in shown in Figure 5B. Homozygous mice failed to respond to the low Cl^-^ solution, indicating a lack of Cl^-^ permeability in the nasal mucosa (Figure 5A and B). Homozygotes also failed to respond significantly to cyclic AMP agonists, adenosine (100 μM) and albuterol (salbutamol, 100 μM) (Figure 5A and B), although a small response was noted a subset of mice (Figure 5B). In contrast, WT mice within the ΔF-CFTR litters responded most vigorously to both the low Cl^-^ maneuver and to the dual cyclic AMP agonists (Figures 5A and B). It should be noted that the inclusion of adenosine was essential for these studies, because Isoprel or salbutamol alone failed to elicit as large or as reproducible responses in the NPD assay. This modification was undertaken based on the work of our colleague and collaborator, Dr. JP Clancy et al., on adenosine regulation of CFTR [50,51]. Notably, the ΔF-CFTR heterozygous mice had an intermediate phenotype between WT mice and homozygous mice with regard to the low Cl^-^ and cyclic AMP-induced responses (Figures 5A and B). Both hyperpolarization responses were significantly less than WT. These *in vivo* NPD data suggest that there is a decrement in CFTR Cl^-^ channel activity in heterozygous ΔF-CFTR carrier mice versus WT mice when assessed across 6 different litters.

**Figure 6:**
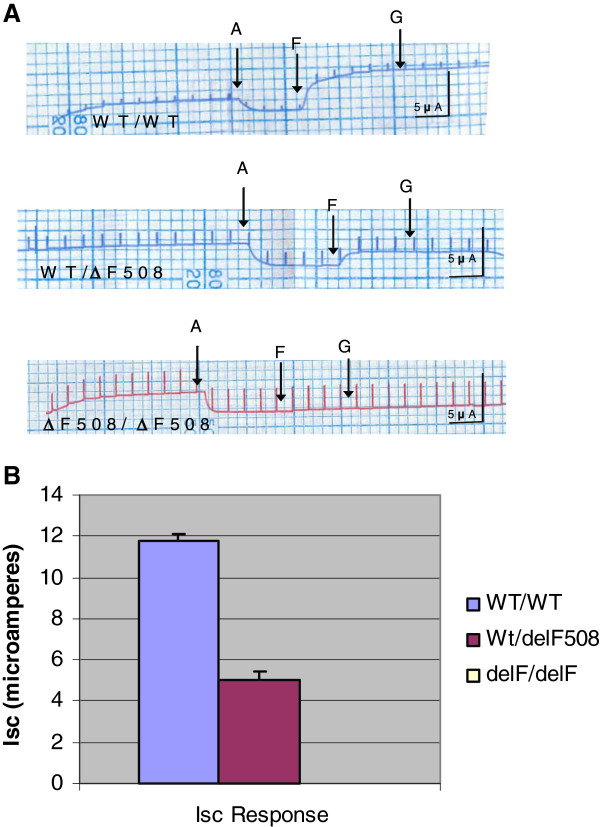
**WT**-**CFTR function is altered by** Δ**F508**-**CFTR in WT**-**CFTR**/**ΔF508**-**CFTR heterozygous MTE A.** Mouse tracheal epithelial cells (MTE) were seeded on Vitrogen coated filter inserts as previously described. On day three, the cells are grown in an air/liquid interface: media on the basolateral side but no media on the apical side. The R_TE_ of the MTE monolayers are allowed to reach 1000 Ω·cm^2^ or above prior to experimentation The MTE monolayer I_SC_ current response to amiloride, forskolin, and genistein are then measured and record ed. **B**. Summary of the I_SC_ data of the three genotypes of the ΔF508 MTE monolayers. Homozygous cell monolayers failed to respond altogether and the summary I_SC_ response was equal to zero.

To derive closely paired *in vitro* data from these litters of ΔF-CFTR mice, tracheae were excised from these same mice in which CFTR NPD measurements were performed previously to isolate and establish mouse tracheal epithelial (MTE) cell monolayers grown on permeable filter supports in primary culture. Typical recordings of I_SC_ from all 3 genotypes from ΔF-CFTR mouse model are shown in Figure 6A. Summary data are shown in Figure 6B. As in *in vivo* NPD assays above, WT MTE monolayers gave the most vigorous response to forskolin and genistein, while heterozygous MTE monolayers responded less well and homozygous ΔF-CFTR MTE monolayers failed to respond altogether (Figure 6A and B). In a subset of recordings, glibenclamide (100 μM) inhibited the CFTR-mediated secretory Cl^-^ current (data not shown). Taken together, these data are similar to results derived from *in vivo* NPD measurements of the same mice and suggest that CFTR activity is partially attenuated in WT/ΔF heterozygous MTE monolayers versus WT/WT controls.

**Figure 7:**
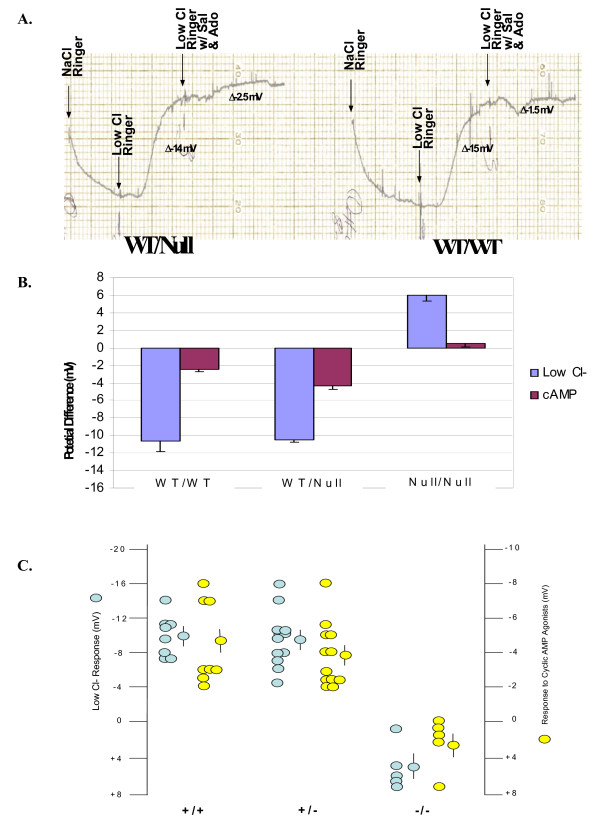
**WT**-**CFTR function is not affected by the null CFTR allele in the Cincinnati WT**-**CFTR**/**Null mice. A**. Nasal Potential Difference Assays (NPD) were performed on the three genotypes of the mice: WT/WT, WT/null, and null/null. NPD recordings were measured for each genotype in the presence of normal Ringer’s solution, low Cl- ringer’s solution, and then low Cl- Ringer’s solution containing the cAMP agonist salbutamol (100 μM) and adenosine (100 μM). **B**. Summary data of the results illustrated in **A**. **C**. Scatterplot which represents the NPD response of each mouse to low Cl- and cAMP agonists segregated to genotype.

Our parallel CF mouse model was the *FABPxCFTR* gut-corrected UNC knockout mouse that remains null for the lung and airways. In this case, the WT controls in these litters have 2 WT *CFTR* alleles, the heterozygous mice have 1 copy of WT *CFTR*, and the homozygous mice are null for *CFTR*. This is an important parallel study to the one above because ΔF-CFTR is not expressed in this mouse model. Figure 7A shows typical NPD recordings from all three *CFTR* genotypes in the Cincinnati bitransgenic mice. In this case, 1 copy of *CFTR* appeared sufficient for full function. There was no difference in low Cl^-^ or cyclic AMP agonist response between the WT and heterozygous mice (Figure 7A,B,C). Homozygous mice failed to respond to either maneuver (Figure 7A,B,C). Figure 7B is a scatterplot which represents the response of each mouse to low Cl^-^ and cAMP agonists segregated to genotype. Results from all mice are shown in the summary data (Figure 7C). These data suggested that 1 *CFTR* allele is enough for full function in an epithelium and in the absence of the ΔF *CFTR* mutation.

**Figure 8:**
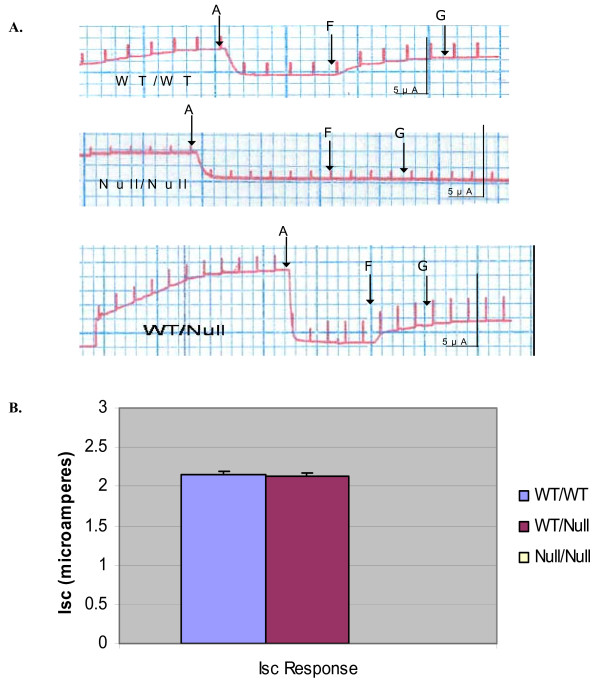
**WT**-**CFTR function is not altered by the null CFTR allele in the Cincinnati WT**-**CFTR**/**null MTE. A.** Mouse tracheal epithelial cells (MTE) were seeded on Vitrogen coated filter inserts as previously described. On day three, the cells are grown in an air/liquid interface: media on the basolateral side but no media on the apical side. The R_TE_ of the MTE monolayers are allowed to reach 1000 Ω·cm^2^ or above prior to experimentation The MTE monolayer I_SC_ current response to amiloride, forskolin, and genistein are then measured and recorded. **B**. Summary of the I_SC_ data of the three genotypes of the bitransgenic MTE monolayers.

We then established MTE monolayers from tracheae of the same mice in the Cincinnati bitransgenic mouse litters. Figure 8A shows representative I_SC_ traces, Figure 8B shows the summary data, and Figure 8C is a scatterplot which represents the response of each mouse to low Cl^-^ and cAMP agonists segregated to genotype. Again, WT and heterozygous MTE monolayers had a similar response to both forskolin and genistein. Homozygous MTE monolayers did not respond to either agonist. Taken together, it is important to note that there is no decrement in overall WT-CFTR function when the number of CFTR alleles is reduced from 2 to 1, suggesting again that ΔF-CFTR is a dominant negative inhibitor of WT-CFTR in airway epithelia.

## Discussion

The results indicate that ΔF-CFTR alters the processing and function of WT-CFTR in a dominant negative manner when co-expressed in a CF human airway epithelial cell. This dominant negative effect required CFTR’s PDZ-binding motif on its C-terminal end. Such an effect of ΔF-CFTR on WT-CFTR could be conferred theoretically by direct protein-protein interaction within a CFTR dimer or multimer, the association of and regulation by accessory proteins (i.e., PDZ binding proteins) for processing, trafficking and function, and/or the association of and regulation by necessary ER chaperones for protein folding.

With these three main biochemical factors impacting upon CFTR biology in native epithelia, we present a single unifying hypothesis to defend this effect in native epithelia. First and foremost, this hypothesis is driven by the fact that our observations hold in native epithelia. Throughout our collective work over the last 15 years, we continue to champion the idea that CFTR functions differently and is processed differently in native human epithelial cell platforms versus non-human or human heterologous cell platforms. CFTR is a limited copy mRNA and protein in native epithelia. Given the copious data on CFTR monomer versus dimer versus larger multimer, we are inclined to agree that CFTR is a monomer; however, that does not mean that CFTR cannot be multimeric in nature. Our central hypothesis speaks to this idea and is predicated on the finding that CFTR resides in a large macromolecular signaling complex that is driven in part by its C-terminal PDZ binding motif. The importance of the PDZ motif has been supported mainly by data generated in native and polarized epithelial cell platforms. There is also evidence in native epithelia for PDZ-interacting proteins being involved in processing and trafficking of CFTR [35-41,53]. Following on these suppositions in a logical manner, ER resident chaperones and the supportive cytoskeleton would be involved actively in the folding and placement of the multiple proteins within this CFTR-resident macromolecular complex. Our hypothesis also assumes that multiple copies (at least 2 copies) of the CFTR protein are processed at the ER, trafficked through the Golgi, and functional at the apical plasma membrane within such a large complex. With similar supportive logic and assuming multiple copies of CFTR per complex and likely multiple complexes within each vesicle as cargo, a ΔF-CFTR copy would attract chaperones that would identify the folding defect and attempt to retain this misfolded ΔF-CFTR protein and associated proteins. More than one ΔF-CFTR protein copy would amplify such attempted ER retention. If copies of WT-CFTR are also present within this large complex, they would be retained, snared or ‘caught up in’ this delF-CFTR retention in other parts of the large complex. Finally, we believe that this dominant negative effect would occur ahead of either Golgi-driven trafficking to the plasma membrane or non-traditional GRASP dependent trafficking that do not involve the complex Golgi apparatus [58,59].

There are a number of proteins that are associated with CFTR that could influence a dominant negative inhibition of WT-CFTR by ΔF-CFTR. Two classes of epithelial-specific accessory proteins likely involved are ER resident chaperones and the PDZ binding proteins. The heat shock family of proteins (HSP) is known to associate with CFTR at the level of the ER as a key group of CFTR chaperones. All members of this family have ATPase activity that is directly linked to their ability to associate/disassociate with their protein substrate. Potential candidates include HSP90, HSP70 and its cognate HSC70 in conjunction with HSP40 and CHIP [60-67]. Recently, Balch and coworkers identified a ‘chaperone trap’ for CFTR that included HSP40, HSP70 and HSP90 [67]. The latter HSP is known to interact with both WT-CFTR and ΔF-CFTR and exists in a dimeric state. A CFTR dimer could conceivably form through an HSP90 dimer at least transiently during CFTR biogenesis in the ER. HSP70 is a less studied protein in CF; however, it does bind CFTR. Its cognate relative, HSC70, is better understood. HSC70 mediates CFTR degradation in the ER through its interaction with HSP40 and CHIP [64]. HSC70’s association with CFTR and other protein substrates is regulated by its fellow chaperone, Hdj-2, an HSP40 family member [64]. In addition, CHIP, as a co-chaperone, binds the HSC70/Hdj-2 complex via one of three tetratricopeptide repeat (TRP) domains. This interaction inhibits the ATPase activity induced by Hdj-2 on HSC 70 and prolongs the interaction of CHIP with HSC70 and with the nascent CFTR peptide. Moreover, CHIP has 3 TRP domains and could bind at least 3 CFTR/HSC70/Hdj-2 complexes [64] and target all to the degradation pathway if one or more of the CFTR polypeptides being processed bore the ΔF-CFTR.

The second class of proteins likely involved in the dominant negative interaction are the PDZ binding domain family of proteins that have the class 1 PDZ domains which recognizes the QDTRL sequence in the end of the CFTR C terminus. Candidates include CAL, EBP50/NHERF-1, E3KARP, and CAP70 that likely influence both trafficking and anchoring of membrane proteins like CFTR and that may be more deeply involved in the ER processing than described previously [35-41,53]. CAL or CFTR-associated ligand is a Golgi resident PDZ protein which can prevent CFTR from reaching the plasma membrane. CAL has only one PDZ domain but exist in a homomultimeric state and could tether multiple CFTR polypeptides together. EBP-50, ezrin-binding protein 50 or Na/H exchange regulatory factor 1 (NHERF-1) has two PDZ binding domains which could, in theory, tether nascent ΔF-CFTR and WT-CFTR polypeptides together if co-expressed in the ER. CAP70, CFTR-associating protein 70, has 4 PDZ domains and 3 of those domains bind CFTR with significant affinity in the order, 3 > 1 > 4. This protein could also bind up to three CFTR molecules and transport them to the cell surface or divert the three “tethered” proteins to the degradation pathway if one or two of the three CFTR polypeptides possessed the ΔF-CFTR mutation. We hypothesize that chaperones, co-chaperones, and PDZ binding proteins resident in the ER may all play a significant role in the ΔF-CFTR/WT-CFTR inhibitory interaction during processing in airway epithelia.

Our finding and the associated CFTR biology in native epithelial cells has profound implications regarding the development of efficient therapeutic methods to correct or replace ΔF-CFTR *in vivo*. Although the understanding of CFTR biology has advanced significantly in recent years, there is much still poorly understood regarding the processing and function of CFTR in native epithelial cells. A primary and fundamental problem which still exists in the field is a lack in the understanding of how epithelial CFTR is processed, what the exact nature of CFTR’s stoichiometry is, and what epithelial accessory proteins interact with epithelial CFTR in the ER, in the Golgi and other organelles, and at the plasma membrane. The discovery and development of CF corrector drugs such as Vertex’s VX-809 being examined in CF clinical trials currently is also influenced by this biology and the concept of a ΔF-CFTR dominant negative inhibition of WT-CFTR when expressed together within an epithelial cell [55,56]. An uncorrected ΔF-CFTR could conceivably still inhibit a corrected ΔF-CFTR in a similar dominant negative manner.

There was a premise within the CF research community that only 10% of cells along the CF airway or a 10% correction of ΔF-CFTR within a given CF cell would be sufficient for a successful therapy. A 10-20% level of correction was achieved with VX-809 in a recent published study [55]. However, VX-809 itself does not appear potent or effective enough as a single drug in recent clinical trials; it was disappointing by itself in a combination trial with the CFTR potentiator drug, VX-770 (ivacaftor, Kalydeco™) in ΔF-CFTR homozygous patients. It is now felt that a 50% level of correction is a better benchmark that is equivalent to 27°C reduced temperature correction in a biochemical correction assay. This also approaches the CF heterozygous condition where a carrier would have 50% of the functional CFTR than a normal or WT individual. This level may need to be the new benchmark for a ΔF-CFTR correction therapy. While many members in the CF field have been resistant to the concept that the CF heterozygote may harbor dysfunction since CF heterozygotes do not display a fully developed CF disease phenotype, correction of a ΔF-CFTR bearing homozygote to a ΔF-CFTR bearing heterozygote would control CF disease. Our work also suggests that more research should be done addressing both the CF patient and the heterozygotic CF carrier in contrast to the normal or non-CF WT controls. One suggestion from these studies is that WT mice, CF heterozygous mice, and CF homozygous mice, especially within ΔF-CFTR mouse models, should be studied as three separate experimental groups in the future. In particular, these three experimental groups may be informative to the study of CFTR biology and its influence on other epithelial cell functions.

## Conclusions

Taken together, ΔF-CFTR inhibition of WT-CFTR during protein processing in the ER of native CF human bronchial epithelial cells explains CF-like disease symptoms but not fully developed CF disease in CF heterozygous carriers, the majority of whom are WT/ΔF carriers in the overall population. Attainment of CF heterozygote level of function with CF corrector drugs and other strategies would also serve as a critical benchmark for CF therapy in the near future. Finally, we propose that both ER-resident chaperones and PDZ-binding proteins likely play critical roles in CFTR-driven multimeric, oligomeric and/or macromolecular complex formation that provide a suitable environment for ΔF-CFTR dominant negative inhibition of WT-CFTR processing and, thus, trafficking and function.

## Competing interests

These studies were purely basic science in nature. There are no competing or non-competing financial interests related to this work.

## Animal ethics

Studies involving animals were performed with UAB CF Center Cores that had and still holds appropriate and approved animal protocols.

## Authors’ contributions

All authors warrant authorship as outlined by BioMedCentral guidelines. TAT was the main driver of this study and made the initial observation of a dominant negative-like effect of delF508-CFTR on wild-type CFTR expression and function when optimizing lipid-based transient transfection methods on polarized and non-polarized airway and other epithelial cell cultures. This study was highly controversial several years ago when it was performed because of the debates over CFTR monomer versus multimer, over disease phenotypes in CF heterozygotes, over differences in CFTR protein processing in epithelial versus heterologous cells, etc. It remains so. AZ assisted TAT on the SPQ and Ussing chamber functional assays. JAF assisted TAT, AZ and EMS on the NPD recordings in the two different CF mouse models on behalf of the UAB CF Center. MD, LF and DMB assisted the program in CF mouse strain breeding and husbandry. EMS and LMS are collaborators and were involved in supervision of the overall study. All authors read and approved the final manuscript.
